# *QuickStats:* Rates of Emergency Department Visits* for Children and Adolescents with Acute Upper Respiratory Infection,^†^ by Age Group — United States, 2021–2022

**DOI:** 10.15585/mmwr.mm7339a5

**Published:** 2024-10-03

**Figure:**
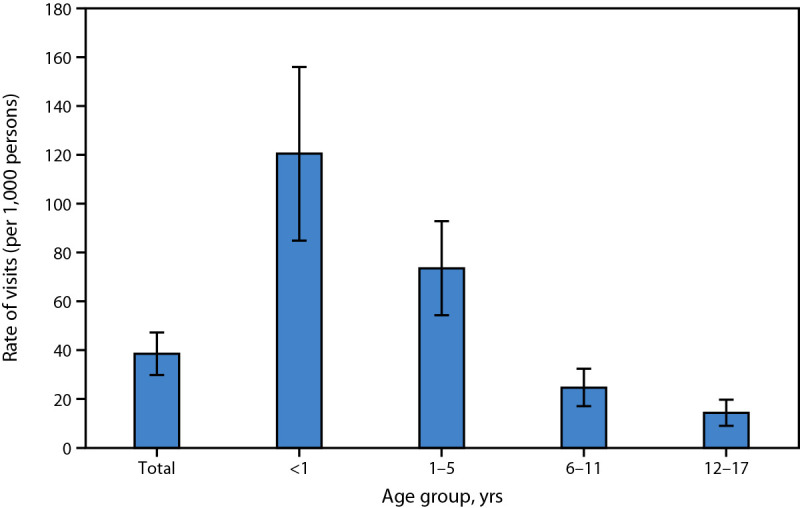
In 2021–2022, the rate for emergency department (ED) visits for children and adolescents with acute upper respiratory infection was 38.6 per 1,000 persons aged <18 years. The ED visit rate was highest for infants aged <1 year (120.5) and decreased by age, with the lowest rate among adolescents aged 12–17 years (14.4).

**Supplementary Table:** https://stacks.cdc.gov/view/cdc/162215

**Source:** National Center for Health Statistics, National Hospital Ambulatory Medical Care Survey, 2021–2022.

For more information on this topic, CDC recommends the following links: https://www.cdc.gov/respiratory-viruses/about/index.html and https://www.cdc.gov/pneumonia/prevention/index.html.

